# Scope and Impact of International Research in Human Pluripotent Stem Cells

**DOI:** 10.1007/s12015-012-9409-0

**Published:** 2012-10-02

**Authors:** Peter Löser, Sabine Kobold, Anke Guhr, Franz-Josef Müller, Andreas Kurtz

**Affiliations:** 1 Robert Koch Institute, DGZ-Ring 1, 13086 Berlin, Germany; 2 Zentrum für Integrative Psychiatrie, Kiel, Germany; 3 Schmerzklinik Kiel, Kiel, Germany; 4 Berlin Brandenburg Center for Regenerative Therapies, Berlin, Germany; 5 Seoul National University, Seoul, Korea

**Keywords:** Human embryonic stem cells (hESC), Human induced pluripotent stem cells (hiPSC), Publication numbers, Impact factors, Citation frequencies

## Abstract

In a recent study published in this journal it was claimed that the rate of publications from US-based authors in the human embryonic stem cell (hESC) research field was slowing or even declining from 2008 to 2010. It was assumed that this is the result of long-term effects of the Bush administration’s funding policy for hESC research and the uncertain policy environment of recent years. In the present study, we analyzed a pool of more than 1,700 original hESC research papers published world-wide from 2007 to 2011. In contrast to the previous study, our results do not support the hypothesis of a decline in the productivity of US-based research but rather confirm a nearly unchanged leading position of US research in the hESC field with respect to both publication numbers and impact of research. Moreover, we analyzed about 500 papers reporting original research involving human induced pluripotent stem cells (hiPSCs) published through 2011 and found a dominant position of US research in this research field as well.

**Electronic supplementary material:**

The online version of this article (doi:10.1007/s12015-012-9409-0) contains supplementary material, which is available to authorized users.

## Introduction

The question how the effects of the federal US funding policy for human embryonic stem cell (hESC) research can be measured is a matter of debate. Whereas some have valued publication numbers as evidence for a growing underperformance of the United States in this research field [1, 2], we observed an unaltered high level of contribution of the United States to hESC research over the last decade [3, 4]. In addition, several studies claimed that the Bush administration’s funding policy was causative for the preferential use of only a few hESC lines, namely Wicell’s H1 and H9 lines [5–7], while we have shown that the preferential use of certain hESC lines is independent of a nation’s stem cell policy [4]. Rather, we found that the current global stem cell usage patterns can be effortlessly explained by a cumulative advantage process independent of restrictive or permissive policy influence, and we showed that this model nearly perfectly mirrors factual worldwide stem cell usage patterns [8].

In a recent paper published in this journal and entitled “The Race Is On: Human Embryonic Stem Cell Research Goes Global”, regional differences in hESC lineage use and a decrease in studies from US-based research groups were reported for a period spanning from 2008 to 2010 [9]. Inter alia it was assumed that lingering effects of the prohibitive stem cell funding policies between 2001 and 2008 and an uncertain policy environment of the first half of the Obama administration was responsible for an observed decrease in productivity of US-based groups in this field. It was stated that more nations joined the hESC “race” in this period of time by “aggressively” publishing in the peer-reviewed literature. Apart from the problem that counting papers published until 2010 might be not a suitable measure for possible effects of the altered stem cell funding policy established in the second half of 2009, we have additional doubts on the conclusions drawn in this study.

While the differences in the regional usage of hESC lines observed by DeRouen and co-workers were already reported before [4], our analysis of a comprehensive dataset on original hESC literature published from 2007 to 2011 shows a different situation for US-based hESC research than reported in the study by DeRouen and co-workers. Our investigation of hESC research papers reveals that both the US share in total numbers of publications produced worldwide and the impact of papers originating from US-based research groups remained nearly unaltered over the last years. In addition, our analysis of human induced pluripotent stem cell (hiPSC) research published through 2011 indicates a moderate decline in the US dominance in this politically non-regulated research field but nevertheless confirms the strong and leading position of US-based hiPSC research.

## Methods

### Data Repositories and Publication Selection

The analyses are based on two literature data banks harbouring 2,407 original research studies involving experimental use of hESCs published from 1998 to 2011 and 514 original hiPSC research papers published from 2007 to 2011, respectively. The data bank on hESC studies was established in 2005 according to the methods reported earlier [10] and also contains information on nearly 1,600 hESC lines and their usage in research based on published data. The hiPSC paper repository was set up in 2009 according to the principles reported recently [11]. Both datasets are updated on an annual basis by manual inspection of large paper pools resulting from the respective searches of the PubMed database accessible through the NIH National Library of Medicine (NIH/NLM). It should be noted, that both repositories only contain studies that report on *original experimental* work on human pluripotent stem cells. Reviews, Comments, Editorials as well as work on ethical and political aspects of research in human pluripotent stem cells are not included. We also did not consider studies, in which only material or cells derived from hESCs or hiPSCs (such as RNA from hESCs or cardiomyocytes derived from hiPSCs) were used or in which data obtained in previous studies (e. g. gene expression data available from the GEO database) were analysed. Publications of protocols that summarize previously reported experimental work were also excluded from our datasets. Assignment of papers to countries was according to the institutional affiliation of the corresponding author.

From 2007 to 2011, 1934 studies involving use of hESCs and 512 hiPSC research papers in line with the outlined criteria were published, respectively. Papers pre-published in 2011 but printed only in 2012 were not included in the analysis. In a portion of these studies both hESCs and hiPSCs were used resulting in an intersection of 392 papers which are present in both datasets. In 174 of those papers hESCs and hiPSCs are either investigated in parallel e. g. to drive conclusions on a broader panel of human pluripotent stem cells [4, 12], or hiPSCs were used to confirm results obtained with hESCs in the same study. However, in the remaining 218 of these studies hESCs were only used for mere comparison with hiPSCs e. g. to verify pluripotency of novel hiPSC lines. These 218 papers were excluded from the analysis of hESC studies giving rise to a pool of 1,716 hESC research papers published from 2007 to 2011 that were analysed in the present study. In case of hiPSC research activities, analysis was started in 2008 so that pioneering work on hiPSCs [13, 14] was omitted. The complete paper list is available on request.

### Determination of Average Impact Factors

The determination of impact factors was performed using the Five Year Impact Factors for 2010 published in the Journal Citation Reports [15]. Of 361 journals that published experimental work on hESCs, 33 did not have an Impact Factor, affecting 45 papers. In case of hiPSC studies, 10 of 123 journals that published experimental work did not have an Impact Factor affecting 12 studies. These 45 and 12 papers, respectively, were not included in the analyses. Consequently, 1,671 hESC and 500 hiPSC studies were analysed for Journal Impact Factors. The Five Year Impact Factor for each journal that had published experimental hESC or hiPSC work, respectively, was multiplied by the number of papers that were published in this journal. The results were added and divided by the total paper numbers to obtain the average Five Year Impact Factors.

### Determination of Average Citation Frequencies

1,289 hESC papers published from 2007 to 2010 and 267 hiPSC research papers published from 2008 to 2010 were analysed for their overall citation frequencies by the end of 2011 using the Scopus database (http://www.scopus.com/home.url). For 4 hESC and 2 hiPSC papers, respectively, no citation frequency could be determined since the journals are not listed in the Scopus database. These papers were omitted from the analysis. Average annual citation frequencies were determined by dividing the citation number by the number of years after the study was published (e. g. for a study that was published in 2007, number of citations from 2008 to 2011 was summated and divided by 4). Citations in the year of publication of a paper (in print) were not considered. Citation analysis was performed in July 2012.

## Results

**Fig. 1:**
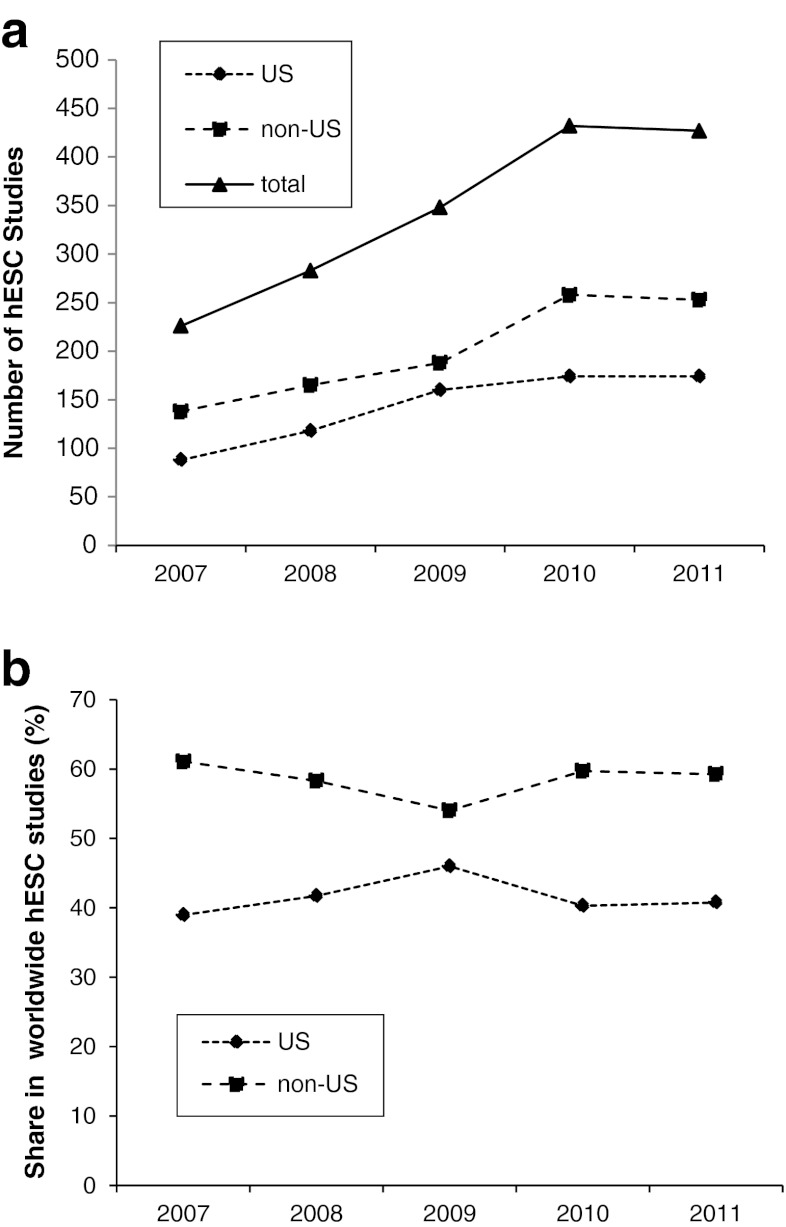
hESC research papers from 2007 to 2011. Shown are the absolute number of hESC research papers (**a**) and the relative share of US and non-US-based studies in the total number of hESC papers (**b**)

Figure 1 shows the results of our analyses of those 1,716 research papers that involved experimental use of hESCs and that where published between 2007 and 2011 in English language journals listed in the Pub Med data base (for details see suppl. Table 1). Studies in which hESCs were used merely for comparison with other human pluripotent stem cells such as hiPSCs were not considered. The number of papers from US-based and non-US groups markedly increased from 2007 to 2011 (Fig. 1a). We observed a slight slowdown in the increase of paper numbers from 2009 to 2011, which might be due to a shift of the scientific interest to hiPSCs in the years after 2007. However, the share in overall publications in hESC remained relatively constant between US- and non-US based groups with a moderate outlier in 2009 (Fig. 1b). The 714 papers published by US-based groups in this five-year period account for about 41.6 % of hESC work published world-wide. This value is nearly identical to that from the previous five-year period (2002 to 2006) when the share of US-based work was 41.2 % (185 of 449 hESC research papers). Thus, our data do not reveal any decline in the contribution of US-based research groups to the relative number of hESC publications. Rather they confirm that the output of research in hESCs has increased both in and outside the United States to a nearly indistinguishable degree.

We also investigated in detail the contribution of groups based in countries with at least 30 research papers in the hESC field from 2007 to 2011 (suppl. Figure 1). Although there was a tendency towards an increase in the number of hESC research studies in most countries from 2007 to 2011, we did not observe an overall relative increase in the percent share of studies from these countries in worldwide publication numbers. For example, whereas the relative share of studies from Japanese groups remained nearly constant, it increased markedly for papers from China and rather decreased for studies from Israel or Singapore in this period of time. It should be noted that in some countries with a rather permissive stem cell policy (such as Israel or Singapore) the absolute research output remained nearly unchanged over the past 5 years. On the other hand, we observed an increase in the number of published studies from Germany which has one of the most restrictive stem cell policies world-wide. In general, the number of countries which are home to research groups publishing results of experimental work involving hESCs increased from 23 in the period 2002 to 2006 to 35 in the 2007 to 2011 period.

**Fig. 2:**
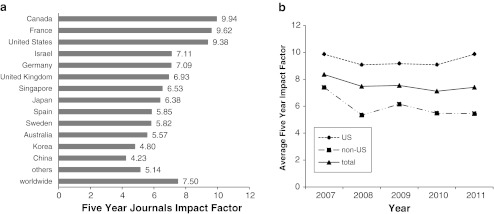
Impact of Journals that published hESC research papers from 2007 to 2011. **a** Overall average Five Year Impact Factors 2010 for hESC research papers published from 2007 to 2011. Data for those 13 countries that contributed more than 30 studies to the hESC research field in this period are shown individually, data for the remaining 22 nations that contributed to hESC research are merged in “others”. **b** Average Five Year Impact Factors 2010 for hESC research papers from US and non-US-based groups for individual years

Although data on publication numbers may provide some insight in past research activities, they do not give a strong indication on the actual impact of this work on international research. We therefore investigated the average Impact Factor of journals that published hESC work from 2007 to 2011 as a further possible proxy for the potential academic impact of a nation’s basic and preclinical hESC research. Figure 2a shows the result of the analysis of those 1,671 hESC research papers that were published from 2007 to 2011 in journals which have an Impact Factor according to the Journal Impact Factors list for 2010. The weighted average Five Year Impact Factor of journals that published hESC work from 2007 to 2011 was about 7.5. However, hESC research studies from groups based in certain countries such as Canada, France and the United States were published in journals with an higher average Five Year Impact Factor (9.94, 9.62 and 9.38, respectively) while papers from groups based in China, Korea or Australia tended to appear in less influential journals (average Five Year Impact Factor 4.23, 4.80 and 5.57, respectively). In the 5 year period analyzed, a moderate decrease in the average Impact Factor of Journals publishing studies from non-US groups was observed, while studies from the United States were published in journals with an nearly constant average Impact Factor (Fig. 2b) underlining the nearly unaltered high visibility of hESC studies from the United States.

**Fig. 3:**
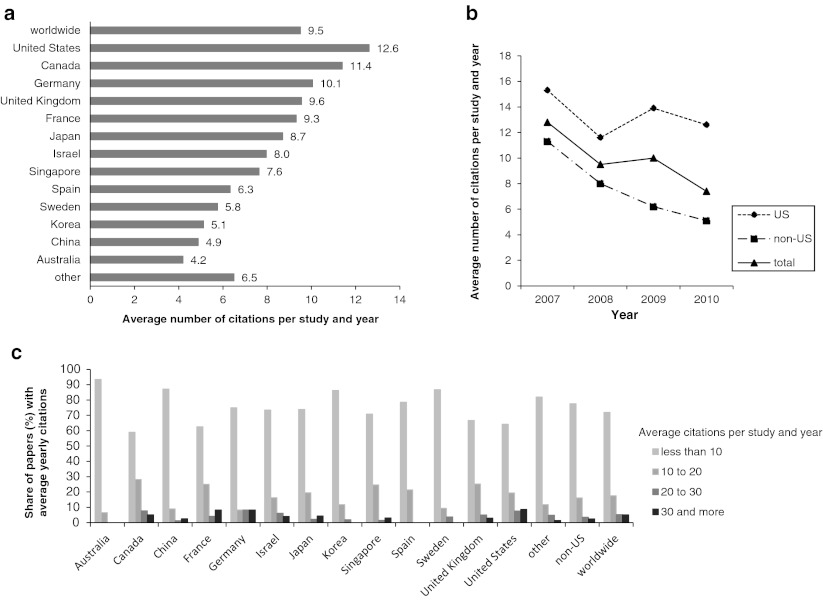
Average annual citation frequencies for hESC research papers published from 2007 to 2010. Citation analysis was performed for the years 2008 to 2011 starting in the year following the publication of a paper. **a** Average annual citation frequencies from 2008 to 2011. **b** Temporal course of average citation frequencies for the years indicated. **c** Share of papers within the indicated range of citations per year in the total number of studies from the given countries. Papers were grouped according to their average annual citation frequency from 2008 to 2011

However, the Impact Factor of a journal does not necessarily reflect the actual influence of each individual study published in this journal. Using the Scopus data base, we therefore analyzed the factual citation numbers until 2011 of hESC research papers published from 2007 to the end of 2010. The high impact of journals publishing US-based hESC research is also reflected in the citation numbers. hESC research papers published from 2007 to 2010 were cited at an average frequency of 9.5 per year. However, studies from the United States were cited at an average of 12.6 times a year, while papers from other countries were cited less frequently (Fig. 3a). Of note, there is only a limited correlation between the average Five Year Impact Factors of journals that published hESC work and the average citation frequencies. In addition to papers from US based groups, only studies from Canada, Germany and the United Kingdom were cited more frequently than the worldwide average. Moreover, when average citation frequencies were investigated on an annual basis, an only moderate decline was observed for studies published by US groups from 2007 to 2010. In sharp contrast, average citation frequencies of studies from other countries declined by more than 50 % from 2007 to 2010 (Fig. 3b). To exclude that the observed diversity in citation frequencies among papers from several nations is due to extremely frequent citation of only a few popular studies we grouped hESC research papers according to their average citation frequency per year (Fig. 3c). The share of papers cited less than 10 times per year was 77.0 % for papers published by non-US groups from 2007 to 2010, and the proportion of papers cited more than 30 times per year was only 2.5 %. In case of papers from groups based in the United States, the share of rarely cited papers was 64.2 %, and papers with an annual citation frequency of 30 or more accounted for 8.7 % of all US studies. Therefore, a rather broad range of hESC papers contributed to the high citation frequency of US-based work. Taken together, our data confirm a relatively constant leadership of US research in the hESC field and strongly argue against the purported decline in the productivity of US based groups in the academic arena.

**Fig. 4:**
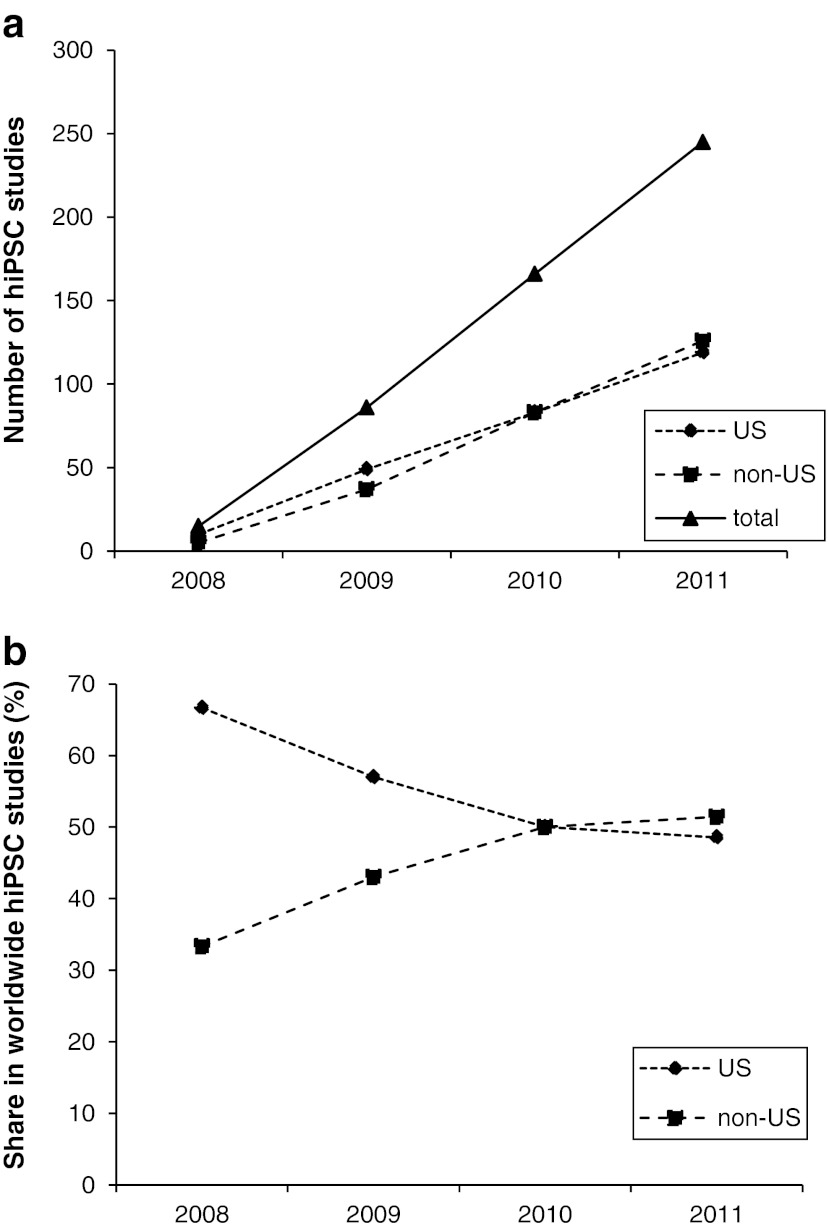
hiPSC research papers from 2007 to 2011. Shown are the absolute number of hiPSC research papers (**a**) and the relative share of US and non-US-based studies in the total number of total hiPSC papers (**b**)

Next, we were interested whether similar or different trends can be observed in the nearly un-regulated field of hiPSC research. We analyzed publications on original work involving hiPSCs that appeared in the past 4 years (2008 to 2011, Fig. 4). By the end of 2011, at least 512 papers describing experimental work involving hiPSCs were available for this period from English language journals indexed in the PubMed data base (for details see suppl. Table 2). A sharp increase in publication numbers was observed from 2008 to 2011, and both US- and non-US-based groups markedly contributed to the elevated research output (Fig. 4a). However, we detected a moderate decrease in the relative share of studies from US-based groups in worldwide publication numbers from 66.7 % in 2008 to 48.6 % in 2011 (Fig. 4b). This might be explained by the early publication of pioneering hiPSC work by several US-based groups which caused a high popularity of this type of research in the following years all over the world. It should be noted that certain countries that significantly contributed to hESC research in the last decade such as Sweden, Singapore, Israel or Korea did not play a major role in hiPSC research by the end of 2011.

**Fig. 5:**
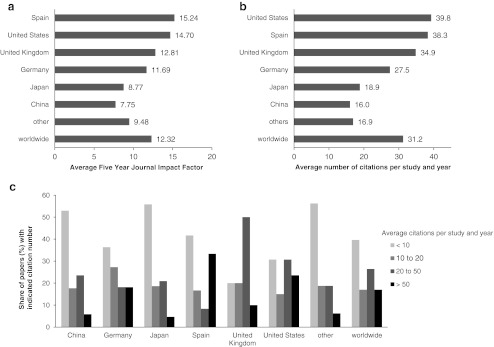
Impact of published hiPSC research. **a** Overall average Five Year Impact Factors 2010 for hiPSC research papers published from 2008 to 2011. Data for those 6 countries that contributed more than 15 studies to the hiPSC research field in this period are shown individually, data for the remaining 14 nations that contributed to hiPSC research are merged in “other”. **b** Average annual citation frequencies for hiPSC research papers published from 2008 to 2010. Citation analysis was performed for the years 2009 to 2011 starting in the year following the publication of a paper. **c** Share of hiPSC research papers within the indicated range of citations per year in the total number of studies from the given countries. Papers were grouped according to their average annual citation frequency from 2009 to 2011

Finally, we performed an analysis of Journal Impact Factors and citation frequencies of hiPSC research papers (Fig. 5). Data for six countries which contributed at least 15 original papers to the hiPSC research field from 2007 to 2011 are shown. Only papers from groups based in Spain were published in journals with a slightly higher average impact factor than US studies (Fig. 5a), while papers published by groups from the United States from 2008 to 2010 were cited most frequently (Fig. 5b). We observed a good general correlation between the average Five Year Impact Factors and the actual citation frequencies. Surprisingly, studies from Japan which is believed to have a leading position in the hiPSC research field seem to be less influential according to both Journal Impact Factors and actual citation frequency, despite the high number of Japanese papers in this field. Again, grouping of papers according to their citation frequency per year revealed, that a major proportion of studies from the United States were cited more than 50 times per year. Thus, a rather broad panel of papers from US groups contributed to the observed high citation rates. In contrast, only few studies from Japan or China are cited as frequently. Thus, although there is a relative decline in output from US-based groups, US based research continues to occupy a leading position in the hiPSC field as well.

## Discussion

There has been some controversy on the effects of regulatory policies on hESC research output and about the reasons for the preferred usage of an only restricted set of hESC lines [2–4, 6, 7, 10, 12]. Most recently, it has been suggested that embargo effects from past restrictive policies in the US may contribute to a reduced output by US-based researchers in recent years, while non-US labs thrive because they were able to freely use any hESC cell line without budget restrictions [9]. Our analysis of the available data did not confirm a reduced relative academic output or impact of US-based hESC research over time.

In fact, our data show that the number of publications in the hESC research field increased globally over the past 5 years, independent of a more or less restrictive stem cell policy or ideology-driven research funding. Although a short-term effect of a restrictive budget policy might have delayed research output in the US after 2001 [16], factors such as general research and alternative budgets, research capacities and human resources or the availability of research material all contribute to the research output and may compensate for politically motivated restrictions. For example, in the United States the state funding for stem cell research partially exceeded federal funding by the NIH in the last years [17]. For the US, our data show no decline in relative contribution to international hESC research as measured in numbers of published research and in terms of its impact, and if anything we found a trend towards a relative increase in the number of citations of work published by US-based researchers as compared to work from researchers based outside the United States. Independent of the US situation with 14 hESC research papers published in 2003 (45.2 % of global output), 41 in 2004 (41.8 %), 49 in 2005 (37.1 %) and 174 in 2011 (40.7 %), hESC research output increased globally with higher numbers of publications, from 31 in 2003 up to newly 430 in 2011.

The reason for the supposed loss of leadership of the United States in hESC research or, more generally, pluripotent stem cell research was blamed on the restrictive funding policy in the US, and its long-term aftereffects. Already in 2006 it was reported that the US share in hESC studies published through 2004 experienced a decline starting in 2001 [1]. Levine identified the United States as an underperformer in hESC research relative to the unrelated RNAi research field [2] although the conclusions of this study have been challenged [3]. A recent citation analysis identified a lag in US production in hESC research of up to 40 % behind anticipated levels when using studies involving RNAi as a reference [16]. However, this lag was most prominent from 2001 to 2003 and although RNAi was – as hESCs – first described in 1998 by a US-based group, studies involving RNAi might not be the best choice for the intended comparison. In contrast to pluripotent stem cells, experimental use of RNAi does neither require a rare and difficult to propagate material (such as stem cells in complex culture systems) nor scientists trained in special experimental skills beyond common molecular biology techniques. In our hands-on experience [18, 19], both factors limited significantly the development of the hESC research field. There is no doubt that restrictions and regulatory uncertainty can have a major impact on researchers [20] and may delay or even prevent scientific progress. However, our analysis of publications does not confirm but clearly refutes the hypothesis of a relative decline of US productivity in hESC research.

It can only be speculated about the reasons for the apparent discrepancy between our data and the recent findings of DeRouen and co-workers. While these authors used a data pool of 2,086 hESC and hiPSC papers published from 1998 to 2010 for their analysis, our dataset for this period of time contains 1940 original research papers. Thus, the paper pool seems to be of a comparable size, and inclusion and exclusion criteria may be the prime reason for different results obtained in both studies. The public availability of the datasets used in the paper of DeRouen et al. as well as in other studies from the same group would have been helpful for understanding obvious discrepancies. In addition, the period of time analyzed by DeRouen and co-workers did only span 3 years and might not be sufficient to draw appropriate conclusions on the principal development of a research field. Moreover, it may be questionable in general whether the number of publications alone is a suitable measure for the impact of a nation’s research. We suggest that taking additional factors such as Journal Impact Factors and citation numbers into account may be more adequate. Doing so, we did not find any evidence for a decline in the US contribution to the hESC field.

For the essentially unrestricted research on hiPSC we found a different outcome with respect to the relative weight of US and global research output. By 2008, neraly 70 % of all hiPSC work was published by US-based groups, but the US share in published work on hiPSCs dropped to below 50 % in 2011. At the same time, US-based research in the hiPSC field maintained a continuing high visibility. It would be premature to conclude that the observed decline in the relative share of US contribution to the hiPSC field may reflect a diminished competitiveness of US research. Rather, we suggest that the undisputable initial dominance of US research in this field was due to pioneering work of several US-based groups which caused a high popularity of hiPSC research in the following years. A reason for the comparatively fast ascent of hiPSC research outside of the US might be that a high number of researchers have been trained by now and are capable to work with human pluripotent stem cells. The easier availability of other resources that are needed for this type of research which were built up during the previous hESC era may also contribute to the rapid development of the hiPSC field. It is surprising, however, that research from Japan performed below average in terms of impact during the reported time frame.

In summary, our data shows an unaltered strong contribution of US research to the hESC field. This may be supportive for the hypothesis that research output is obviously not severely hampered by budgetary regulation as long as alternative budget options are available [16]. Moreover, research on hiPSCs is characterized by a moderate decrease in US dominance in this field with regard to paper numbers, while the impact of US research remains at a high level. Factors such as increasing international collaboration, publication bias or distribution of resources may have specific effects on this type of research and require further investigation.

## Electronic supplementary material

Below is the link to the electronic supplementary material.

Suppl. Figure 1 Detailed analysis of hESC research papers from groups residing outside the United States. Studies from countries that contributed more than 30 hESC research papers to the field from 2007 to 2011 were included. Shown are the absolute number of hESC research papers (a) and the relative share of studies in the total number of (world-wide) hESC papers (b). (PPTX 66 kb)

Suppl. Table 1 Numbers of papers reporting original work on hESCs. Assignment of a paper to a specific country was performed according to the academic affiliation of the corresponding author. Note that studies in which hESCs were used for mere comparison with hiPSCs were not included. (DOCX 22 kb)

Suppl. Table 2 Numbers of papers reporting original experimental work involving hiPSCs. Assignment of a paper to a specific country was performed according to the academic affiliation of the corresponding author. (DOCX 19 kb)
