# Supplementary material for: Scope and Impact of International Research in Human Pluripotent Stem Cells
Source: Stem Cell Rev. 2012 Oct 2;8(4):1048–55. doi: 10.1007/s12015-012-9409-0 (PMC3505517; doi:10.1007/s12015-012-9409-0)
Supplement: Supplementary file 1 — Detailed analysis of hESC research papers from groups residing outside the United States. Studies from countries that contributed more than 30 hESC research papers to the field from 2007 to 2011 were included. Shown are the absolute number of hESC research papers (a) and the relative share of studies in the total number of (world-wide) hESC papers (b). (PPTX 66 kb) [file 12015_2012_9409_MOESM1_ESM.pptx]

## Slide 1
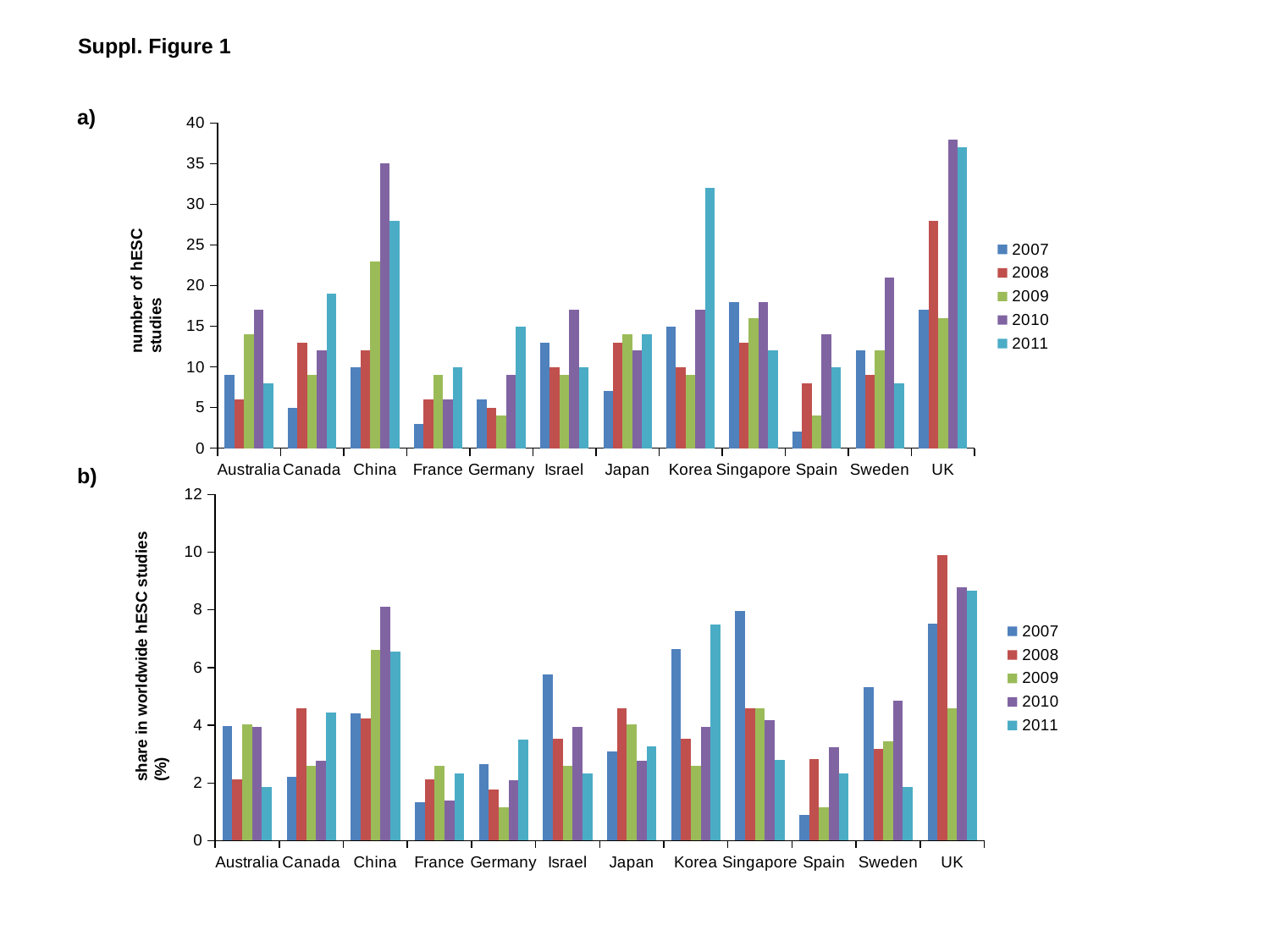

Suppl. Figure 1
a)
### Chart
| Category | 2007 | 2008 | 2009 | 2010 | 2011 |
|---|---|---|---|---|---|
| Australia | 9.0 | 6.0 | 14.0 | 17.0 | 8.0 |
| Canada | 5.0 | 13.0 | 9.0 | 12.0 | 19.0 |
| China | 10.0 | 12.0 | 23.0 | 35.0 | 28.0 |
| France | 3.0 | 6.0 | 9.0 | 6.0 | 10.0 |
| Germany | 6.0 | 5.0 | 4.0 | 9.0 | 15.0 |
| Israel | 13.0 | 10.0 | 9.0 | 17.0 | 10.0 |
| Japan | 7.0 | 13.0 | 14.0 | 12.0 | 14.0 |
| Korea | 15.0 | 10.0 | 9.0 | 17.0 | 32.0 |
| Singapore | 18.0 | 13.0 | 16.0 | 18.0 | 12.0 |
| Spain | 2.0 | 8.0 | 4.0 | 14.0 | 10.0 |
| Sweden | 12.0 | 9.0 | 12.0 | 21.0 | 8.0 |
| UK | 17.0 | 28.0 | 16.0 | 38.0 | 37.0 |number of hESC studies
b)
### Chart
| Category | 2007 | 2008 | 2009 | 2010 | 2011 |
|---|---|---|---|---|---|
| Australia | 3.982300884955752 | 2.1201413427561837 | 4.022988505747127 | 3.935185185185185 | 1.873536299765808 |
| Canada | 2.2123893805309733 | 4.593639575971731 | 2.586206896551724 | 2.7777777777777777 | 4.449648711943794 |
| China | 4.424778761061947 | 4.240282685512367 | 6.609195402298851 | 8.101851851851851 | 6.557377049180328 |
| France | 1.3274336283185841 | 2.1201413427561837 | 2.586206896551724 | 1.3888888888888888 | 2.3419203747072603 |
| Germany | 2.6548672566371683 | 1.76678445229682 | 1.1494252873563218 | 2.083333333333333 | 3.51288056206089 |
| Israel | 5.752212389380531 | 3.53356890459364 | 2.586206896551724 | 3.935185185185185 | 2.3419203747072603 |
| Japan | 3.0973451327433628 | 4.593639575971731 | 4.022988505747127 | 2.7777777777777777 | 3.278688524590164 |
| Korea | 6.637168141592921 | 3.53356890459364 | 2.586206896551724 | 3.935185185185185 | 7.494145199063232 |
| Singapore | 7.964601769911504 | 4.593639575971731 | 4.597701149425287 | 4.166666666666666 | 2.810304449648712 |
| Spain | 0.8849557522123894 | 2.8268551236749118 | 1.1494252873563218 | 3.2407407407407405 | 2.3419203747072603 |
| Sweden | 5.3097345132743365 | 3.180212014134275 | 3.4482758620689653 | 4.861111111111112 | 1.873536299765808 |
| UK | 7.52212389380531 | 9.89399293286219 | 4.597701149425287 | 8.796296296296296 | 8.665105386416862 |share in worldwide hESC studies (%)
