# Supplementary material for: Scope and Impact of International Research in Human Pluripotent Stem Cells
Source: Stem Cell Rev. 2012 Oct 2;8(4):1048–55. doi: 10.1007/s12015-012-9409-0 (PMC3505517; doi:10.1007/s12015-012-9409-0)
Supplement: Supplementary file 2 — Numbers of papers reporting original work on hESCs. Assignment of a paper to a specific country was performed according to the academic affiliation of the corresponding author. Note that studies in which hESCs were used for mere comparison with hiPSCs were not included. (DOCX 22 kb) [file 12015_2012_9409_MOESM2_ESM.docx]

Suppl. Table 1: Numbers of papers reporting original work on hESCs. Assignment of a paper to a specific country was performed according to the academic affiliation of the corresponding author. Note that studies in which hESCs were used for mere comparison with hiPSCs were not included.

| Country | total | 2007 | 2008 | 2009 | 2010 | 2011 |
| --- | --- | --- | --- | --- | --- | --- |
| Argentinia | 1 | 0 | 0 | 0 | 0 | 1 |
| Australia | 54 | 9 | 6 | 14 | 17 | 8 |
| Austria | 2 | 0 | 0 | 1 | 0 | 1 |
| Belgium | 21 | 1 | 4 | 7 | 3 | 6 |
| Brazil | 3 | 0 | 0 | 1 | 0 | 2 |
| Canada | 58 | 5 | 13 | 9 | 12 | 19 |
| China | 107 | 9 | 12 | 23 | 35 | 28 |
| Czech Republic | 11 | 0 | 3 | 3 | 3 | 2 |
| Denmark | 14 | 2 | 2 | 4 | 3 | 3 |
| Estonia | 2 | 0 | 1 | 0 | 0 | 1 |
| Finland | 27 | 3 | 2 | 7 | 9 | 6 |
| France | 34 | 3 | 6 | 9 | 6 | 10 |
| Germany | 39 | 6 | 5 | 4 | 9 | 15 |
| Hungary | 6 | 0 | 1 | 1 | 1 | 3 |
| India | 20 | 5 | 4 | 6 | 4 | 1 |
| Iran | 15 | 1 | 1 | 3 | 4 | 6 |
| Israel | 59 | 13 | 10 | 9 | 17 | 10 |
| Italy | 15 | 5 | 2 | 5 | 2 | 1 |
| Japan | 60 | 7 | 13 | 14 | 12 | 14 |
| Korea | 83 | 15 | 10 | 9 | 17 | 32 |
| Malaysia | 4 | 0 | 0 | 0 | 2 | 2 |
| Netherlands | 21 | 2 | 6 | 2 | 6 | 5 |
| New Zealand | 1 |  | 0 | 0 | 1 | 0 |
| Norway | 1 | 0 | 0 |  | 1 | 0 |
| Portugal | 4 | 0 | 0 | 1 | 1 | 2 |
| Romania | 1 | 0 | 0 | 0 | 0 | 1 |
| Russia | 10 | 1 | 2 | 2 | 1 | 4 |
| Singapore | 77 | 18 | 13 | 16 | 18 | 12 |
| Spain | 38 | 2 | 8 | 4 | 14 | 10 |
| Sweden | 62 | 12 | 9 | 12 | 21 | 8 |
| Switzerland | 10 | 1 | 2 | 4 | 0 | 3 |
| Thailand | 1 | 0 | 0 | 1 | 0 | 0 |
| Turkey | 5 | 1 | 2 | 1 | 1 | 0 |
| United Kingdom | 136 | 17 | 28 | 16 | 38 | 37 |
| United States | 714 | 88 | 118 | 160 | 174 | 174 |
| total | 1716 | 226 | 283 | 348 | 432 | 427 |
