# Supplementary material for: Scope and Impact of International Research in Human Pluripotent Stem Cells
Source: Stem Cell Rev. 2012 Oct 2;8(4):1048–55. doi: 10.1007/s12015-012-9409-0 (PMC3505517; doi:10.1007/s12015-012-9409-0)
Supplement: Supplementary file 3 — Numbers of papers reporting original experimental work involving hiPSCs. Assignment of a paper to a specific country was performed according to the academic affiliation of the corresponding author. (DOCX 19 kb) [file 12015_2012_9409_MOESM3_ESM.docx]

Suppl. Table 2: Numbers of papers reporting original experimental work involving hiPSCs. Assignment of a paper to a specific country was performed according to the academic affiliation of the corresponding author.

| Country | total | 2008 | 2009 | 2010 | 2011 |
| --- | --- | --- | --- | --- | --- |
| AUSTRALIA | 7 | 0 | 0 | 2 | 5 |
| BRAZIL | 3 | 0 | 0 | 0 | 3 |
| CANADA | 10 | 0 | 3 | 0 | 7 |
| CHINA | 32 | 2 | 5 | 10 | 15 |
| FINLAND | 3 | 0 | 0 | 1 | 2 |
| FRANCE | 8 | 0 | 1 | 2 | 5 |
| GERMANY | 22 | 0 | 3 | 8 | 11 |
| IRAN | 8 | 0 | 1 | 4 | 3 |
| ISRAEL | 13 | 0 | 1 | 5 | 7 |
| ITALY | 2 | 0 | 0 | 0 | 2 |
| JAPAN | 78 | 2 | 14 | 27 | 35 |
| KOREA | 9 | 0 | 0 | 5 | 4 |
| NETHERLANDS | 2 | 0 | 0 | 1 | 1 |
| ROMANIA | 1 | 0 | 0 | 0 | 1 |
| RUSSIA | 4 | 0 | 0 | 1 | 3 |
| SINGAPORE | 8 | 0 | 0 | 2 | 6 |
| SPAIN | 17 | 1 | 4 | 7 | 5 |
| SWEDEN | 2 | 0 | 0 | 2 | 0 |
| SWITZERLAND | 3 | 0 | 1 | 0 | 2 |
| UNITED KINGDOM | 19 | 0 | 4 | 6 | 9 |
| UNITED STATES | 261 | 10 | 49 | 83 | 119 |
| total | 512 | 15 | 86 | 166 | 245 |
